# Combining Microbial Cellulose with FeSO_4_ and FeCl_2_ by Ex Situ and In Situ Methods

**DOI:** 10.3390/polym17131743

**Published:** 2025-06-23

**Authors:** Silvia Barbi, Marcello Brugnoli, Salvatore La China, Monia Montorsi, Maria Gullo

**Affiliations:** 1 Department of Sciences and Methods for Engineering, University of Modena and Reggio Emilia, Via Amendola 2, 42122 Reggio Emilia, Italy; monia.montorsi@unimore.it; 2 Department of Life Sciences, University of Modena and Reggio Emilia, Via Amendola 2, 42122 Reggio Emilia, Italy; salvatore.lachina@unimore.it (S.L.C.); maria.gullo@unimore.it (M.G.); 3 EN&TECH—Interdepartmental Center for Industrial Research and Technology Transfer in the Field of Integrated Technologies for Sustainable Research, Efficient Energy Conversion, Energy Efficiency of Buildings, Lighting and Home Automation, University of Modena and Reggio Emilia, Via Amendola 2, 42122 Reggio Emilia, Italy

**Keywords:** *Komagataeibacter*, bacterial cellulose, sustainable electronics, iron composites, iron gluconate

## Abstract

Environmentally sustainable methods for producing flexible electronics, such as paper-based energy harvesters in nanogenerators, are a major objective in materials science. In this frame, the present study investigated two different *Komagataeibacter* sp. strains (K2G30 and K2G44), never tested as biocatalysts for the production of bacterial cellulose (BC) functionalized with iron particles to provide potential electrical conductivity. Two functionalization strategies (ex situ and in situ) were evaluated using two iron compounds FeCl_2_ and FeSO_4_, individually and in combination (up to 0.1% *w*/*v*), to assess efficiency and feasibility. In addition, a Design of Experiment approach was implemented to calculate quantitative mathematical models to correlate the functionalization methods with the iron amount in the BC. Among the tested conditions, BC produced by strain K2G44 using the ex situ method with FeCl_2_ showed the most promising results, achieving the highest iron content (~37% atomic weight) with a highly homogeneous dispersion of iron nanoparticles. Moreover, the in situ BC functionalization using FeSO_4_ led to the formation of iron gluconate. FeSO_4_ alone significantly enhanced BC production in the in situ process, with yields of 2.62 ± 0.15 g/L for K2G30 and 2.05 ± 0.09 g/L for K2G44.

## 1. Introduction

Bacterial cellulose (BC) in its native and functionalized forms offers a promising perspective for the enhancement of sustainable manufacturing processes. In the past decade, advances in studying BC production have led to many applications, including biomedical, pharmaceutical, food, textiles, and others [1,2,3]. BC possesses an ultrafine fibrous structure, a high surface area, and is highly porous, thus allowing it to absorb both liquids and small molecules. It contains OH groups on the surface, which allow the interaction with a variety of functional groups, thus leading to the formation of BC-based functional materials for different applications [2].

The approaches to biofabricate functionalized BC encompass different ex situ and in situ strategies [4]. The ex situ approach consists of impregnating BC films of the functional material after pure BC is formed and harvested. Functionalization can occur through physical or chemical modifications, by physical adsorption and chemical reactions, respectively. Owing to the BC porous matrix nature, ex situ physical modifications are extremely versatile and exploited. Indeed, BC pores can be filled with materials or solutions, and the presence of hydroxyl groups of cellulose chains results in strong hydrogen bonds between BC molecules and the material [5]. On the other hand, the in situ approach consists of supplementing the functionalizing compounds in the culture medium where the bacterial culture is inoculated. Through this approach, the compound is directly included in the BC during its biosynthesis. Several polymers, proteins, metals, nanoparticles, or antimicrobial compounds have been reported to be successfully incorporated into BC [5,6,7,8]. For example, recent research indicates the feasibility of lignin (a waste byproduct in the paper industry) to enhance the mechanical strength, toughness, elongation, and thermal stability of BC after in situ functionalization [9,10]. Again, TEOS (Tetraethyl orthosilicate) has been successfully included in BC with a favorable increase in tensile strength [11]. Moreover, the latest studies report the feasibility of microbial co-culture systems to functionalize BC with specific microbial compounds synthesized by bacteria [12,13,14].

BC-based materials have shown promising results in the development of flexible electronic devices. Lei et al. [15] and Sun et al. [16] both highlighted the potential for a stretchable substrate and a high-performance green electronic substrate, respectively. Kim et al. [17] and Jeon et al. [18] both explored the use of BC in biomedical applications, focusing on the production of tissue engineering scaffolds and wound dressing materials [17] and an electro-active biopolymer actuator for biomedical applications [18]. In addition, specifically paper-based energy harvesting nanogenerators are one of the arising technologies to exploit the peculiar properties of BC to substitute traditional but not biodegradable materials such as polytetrafluoroethylene (PTFE), fluorinated ethylene propylene (FEP), and polydimethylsiloxane (PDMS) [19]. Despite great expectations, several key challenges remain pivotal to unlocking the full potential of functionalized BC, among those: complex fabrication and limited scalability for cost-effective production [20,21].

Song et al. [22] further demonstrated the flexibility of BC in the fabrication of soft, permeable microcapsules, which could have potential applications in substance encapsulation and protection. Among these categories of hybrid materials for electronics, BC-based metal complexes have found many applications [23,24]. In particular, some studies have explored the properties and applications of BC-iron composites. Zeng et al. [25] observed a further enhancement of the BC functionality by fabricating nanocomposite films with iron oxide nanoparticles, exhibiting superparamagnetic behavior and flexibility. Kong and Wilson [26] found that the coating efficiency of iron oxide onto BC is influenced by the accessibility of the biopolymers’ -OH groups. Calvini and Silveira [27] observed that iron-treated cellulose papers become highly hydrolyzed and oxidized over time, with excess iron leading to the formation of iron (II) oxalate. However, most of the studies focused on the exploitation of the ex situ approach to functionalize the BC, involving immersion and co-precipitation with sodium hydroxide [28] or ammonia [23,24,29].

Acetic acid bacteria (AAB) are aerobic, oxidative bacteria well known to convert ethanol and sugars into the corresponding oxidized products. The main metabolites deriving from the oxidative pathway of AAB are acetic acid and gluconic acid, produced from ethanol and glucose, respectively [30]. Moreover, among AAB, the production of exopolysaccharides like BC and levan is well known, especially within species of the genera *Komagataeibacter* and *Novacetimonas*. Intra- and interspecies differences in BC synthesis are also documented. *K. xylinus* has a peculiar cellulose synthesis machinery that makes it the model organism for BC synthesis. Other species, like *K. rhaeticus* and *K. kombuchae*, *K. pomaceti*, *N. hansenii*, and *N. cocois* produce BC at different yields [1,31].

In this study, we examined the influence of iron precursor type and functionalization strategy on the modification of BC. Specifically, we evaluated both ex situ and in situ functionalization approaches using FeSO_4_ and FeCl_2_ as iron sources. Considering the consolidated literature, the in situ method is also the least explored, with a very limited number of articles that consider comparison among in situ and ex situ approaches [32]. A key novelty of this work lies in the use of two previously unexplored *Komagataeibacter* sp. strains, K2G30 and K2G44, for both BC production and functionalization—an aspect not addressed in prior literature. The study was based on the hypothesis that these two strains, which differ in their inherent BC productivity [33], could generate composite materials with distinct morphology and functional properties. To validate this, we utilized a multidisciplinary methodology integrating morphological and chemical characterization techniques (ESEM, XRD, and FT-IR) with a statistically driven rational approach (Design of Experiments and Analysis of the Variance). This approach enabled the development of predictive models for key output variables, such as iron content, as a function of critical process parameters, including bacterial strain and iron precursor type. As far as we know, this is the first study that implements a statistical approach to collect and analyze the experimental data on this topic. The outcomes demonstrated that both functionalization strategies are effective but yield distinct characteristics depending on the strain and conditions used. The observed differences highlighted the potential of strain-specific optimization in tailoring Fe-BC composites for diverse technological features, broadening the areas of application.

## 2. Materials and Methods

### 2.1. Bacterial Strains and Cultivation Conditions

Two AAB strains deposited at the UMCC culture collection (Unimore Microbial Culture Collection, Reggio Emilia, Italy), namely K2G30 (UMCC 2756) and K2G44 (UMCC 2972), were used in this study. Details regarding the isolation source and the complete characterization of these two strains can be found elsewhere in the literature [33]. The strains were rehydrated from −80 °C storage conditions by cultivation in Hestrin-Schramm (HS) medium (20 g/L glucose anhydrous, 10 g/L yeast extract, 5 g/L polypeptone, 2.7 g/L disodium phosphate anhydrous, and 1.15 g/L citric acid monohydrate). Cultures were incubated aerobically in static conditions for 4 days at 28 °C.

### 2.2. Ex Situ Functionalization

To reduce the possible number of tests given by the simultaneous evaluation of the different experimental variables (e.g., bacterial strains, sonication time, and chemical formulation of the functionalization solution) a Design of Experiments approach was employed. A combined mixture design was implemented (Table 1) to simultaneously evaluate the proportion of the two functionalization reagents and other environmental variables (bacterial strain and sonication time). The software used for the experimental plan design was Design Expert 13—Statease (USA). A total of 19 samples were therefore prepared following Table 1, including repetition and central point. Tests were performed in a randomized order with respect to each variable variation to reduce possible effects of uncontrolled external variables. For each one of the tests proposed in Table 1, BC produced in HS medium, according to the previous procedure, was first washed in distilled water and then immersed in a water solution (0.1 *w*/*v*) containing FeCl_2_ and FeSO_4_ at different ratios for 1 h at 60 °C. Afterward, samples were subjected to sonication as shown in Table 1, through a digital sonicator (DU-32, Argolab, Carpi, Italy) to promote the homogenous dispersion of the iron particle into the fibrous structure of BC, also preventing the formation of agglomerates. The treated BC samples were dried at room temperature (23 °C) until constant weight was reached.

### 2.3. In Situ Functionalization

An aliquot of pre-inoculum obtained from K2G30 and K2G44 (5% *v*/*v*) was transferred to a 100-mL Erlenmeyer flask containing HS broth and cultivated aerobically in static conditions, for 4 days at 28 °C. Prior to the inoculation in the Fe-enriched medium, bacterial cells were removed from the spent medium (HS broth) by centrifugation at 8000× *g* for 5 min. The pellets were resuspended in HS media enriched with FeSO_4_ and FeCl_2_ (Sigma Aldrich, Burlington, MA, USA, purity > 99.9%) at different concentrations. Specifically, for the in situ modification, each bacterial strain was cultivated in four Fe-enriched HS media, designed to include (i) 03% FeSO_4_-0.02% FeCl_2_ *w*/*v*, (ii) 0.04% FeSO_4_-0.01% FeCl_2_ *w*/*v*, (iii) FeSO_4_ at 0.05% *w*/*v*, and (iv) FeSO_4_ at 0.10% *w*/*v*. Bacterial cultures were cultivated at 28 °C for 4 days with an inoculum volume set at 5% *v*/*v*. At the end of the incubation period, flasks were manually shaken and placed in a sonicator (Sonomatic, Langford, BC, Canada) for 10 min to promote well-dispersion of adsorbed and non-adsorbed Fe nanoparticles. Then, BC pellicles were collected and treated in a NaOH 0.1 M solution for 30 min at 80 °C. Further, the pellicles were washed with distilled water, dried at 20 °C, until a constant weight was reached, and weighed afterward (Gibertini E42S, Milan, Italy). Six replicates were prepared for each condition.

### 2.4. Characterizations

Instrumental characterizations were performed on dried BC after functionalization (at 20 °C until constant weight was reached) on all the obtained samples. Structural analysis through IR spectroscopy (FT-IR) was performed with the FTIR VERTEX 70 spectrophotometer (Bruker). Morphological and semi-quantitative chemical analysis was performed through scanning electron microscopy (ESEM) with ESEM Quanta 200 (Fei Company—Oxford Instruments) equipped with an EDS detector for semi-quantitative chemical analysis for the Fe content estimation. Semi-quantitative chemical analysis was performed on 5 different areas of each sample, and the presented numerical results are the average values among these 5 observations of the same sample. Furthermore, a panel test evaluation was conducted on the ex situ sample to identify the physical quality of the dried BC after functionalization, in terms of the homogeneity of the specimen and capability to be detached from the support; the related response is “Film Quality”. The value of this response goes from 1 to 5, where the first is the value related to the worst condition (inhomogeneous sample and very difficult to detach from the drying support), and the latter value is attributed to the best condition (totally homogenous sample and very easy to detach from the support). The most promising samples in terms of quantity of Fe, evaluated through EDS, were also analyzed by X-ray diffraction (XRD, Empyrean, Malvern Panalytical, UK) in bulk (fragment 30 mm × 20 mm) to assess the generated crystalline phases. Data were collected employing the CuKα radiation (λ = 1.54 Å) in Bragg-Brentano mode; the scans were processed in the angular range 5–60° 2θ, with a step size of 0.008° 2θ and scan step time of 30.48 s. Finally, electrical resistivity was assessed with a digital multimeter (Keithley, 2100, Beaverton, OR, USA) and the reported data are the average value of 10 measurements obtained by placing the electrodes at a distance of 1.5 cm.

### 2.5. Molecular Identification of Acetic Acid Bacteria

Genomic DNA extraction was conducted on culture obtained in HS medium, incubated aerobically for 5 days at 28 °C. Moreover, 8 mL of cell cultures were centrifuged at 10,000× *g*, at 4 °C for 10 min. The extraction was performed according to Gullo et al. [33,34]. gDNA concentration was evaluated by NanoDrop™ 1000 Spectrophotometer (Thermo Fisher Scientific, Waltham, MA, USA), while the quality was determined by gel electrophoresis on 1% gel agarose in 1X TBE buffer. Band sizes were determined using a 100 bp plus DNA ladder (Invitrogen, Carlsbad, CA, USA). PCR reaction was performed using primers 27F (AGRGTTYGATYMTGGCTCAG) and 1490R (TACGGYTACCTTGTTACGACTT). The amplified product was purified using the kit DNA Clean & Concentrator™-5 (ZymoResearch) and automated sequencing (Sanger sequencing technique, BIOFAB). The Ab1 file was processed using BioEdit version 7.7.1.0 [35] for end-trimming according to primer length and base quality control. The blast algorithm was used to align the high-quality sequence against the 16S rRNA database. The type strains 16S rRNA sequences of the identified bacterial species were downloaded from the LPNS database 2 [36]. Reference and sequenced 16S sequences were aligned all-vs-all using Muscle v5 [37]. The 16S rRNA sequence was deposited in Genbank under the accession number PP755334. The multiple alignments were imported into MEGA v11.0.13 and trimmed to the same length. A neighbor-joining phylogenetic tree based on 1000 replicates was computed by applying the Tamura-nei DNA evolutionary model [38]. To model evolutionary rate differences among the sequence sites, a discrete Gamma distribution was used. The interactive Tree of Life (ITOL) v6.8.1 was used to visualize the obtained Newick file [39].

### 2.6. Statistical Data Analysis

Data were analyzed through multivariate analysis of the variance (ANOVA), having as a threshold a *p*-value equal to 0.05. The software employed is the same as that already used for experimental plan design. BC yield data were analyzed using R v 4.2.3 [40] at a significance level of *p* < 0.05 using one-way ANOVA. The Tukey post hoc test was used to determine statistical differences among samples.

## 3. Results

### 3.1. Ex Situ Functionalization

Table 1 shows the data collected as responses for the experimental plan (Film quality and Fe%). The data suggest that, in all the samples obtained with the ex situ method, a relevant quantity of Fe (atomic%) was detected. In addition, from a statistical point of view, a sufficient dispersion of the obtained data (e.g., Fe content has its variability from 19.45% to 37.52%) recommends that a statistical evaluation is needed to assess the influence of the input variables on the two responses.

**Figure 1:**
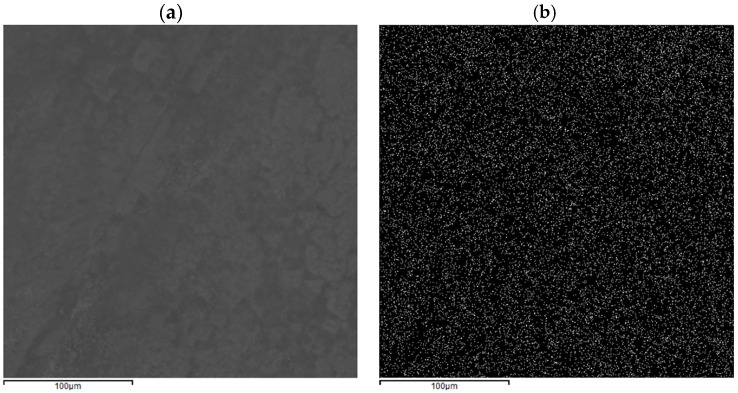
Elemental map of sample RUN 5: (**a**) sample morphology (**b**) Fe elemental distribution.

**Figure A1:**
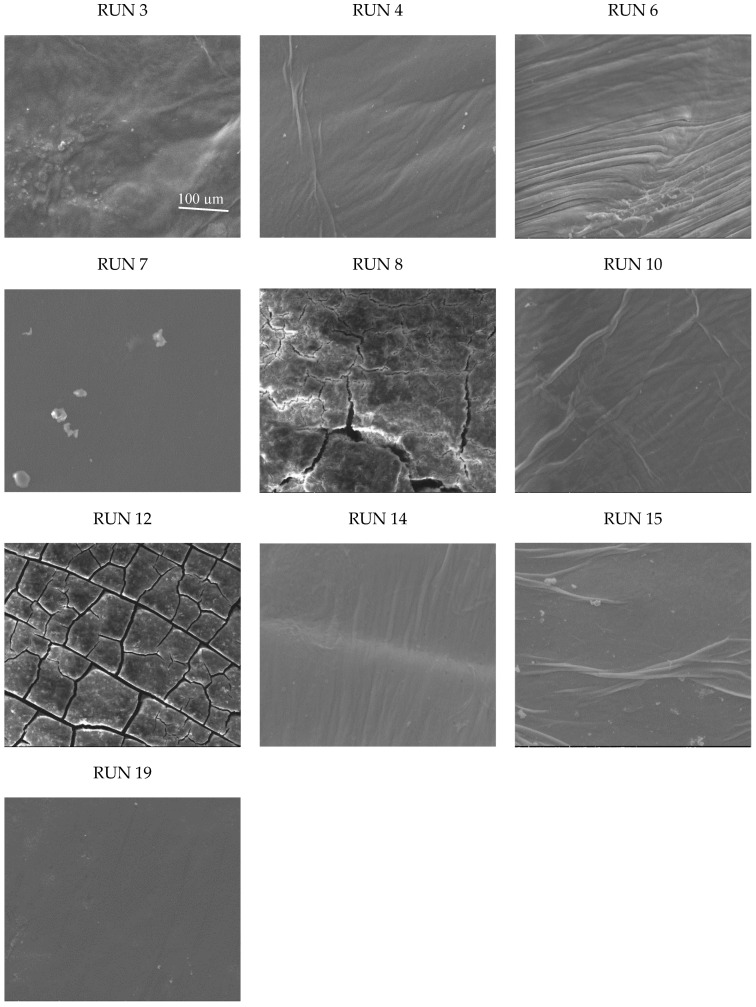
ESEM micrographs at 1000× (ETD detector) of the samples obtained from K2G30 functionalized with trough ex situ method (the scale bar indicated in the first micrograph can be applied for all the others).

**Figure A2:**
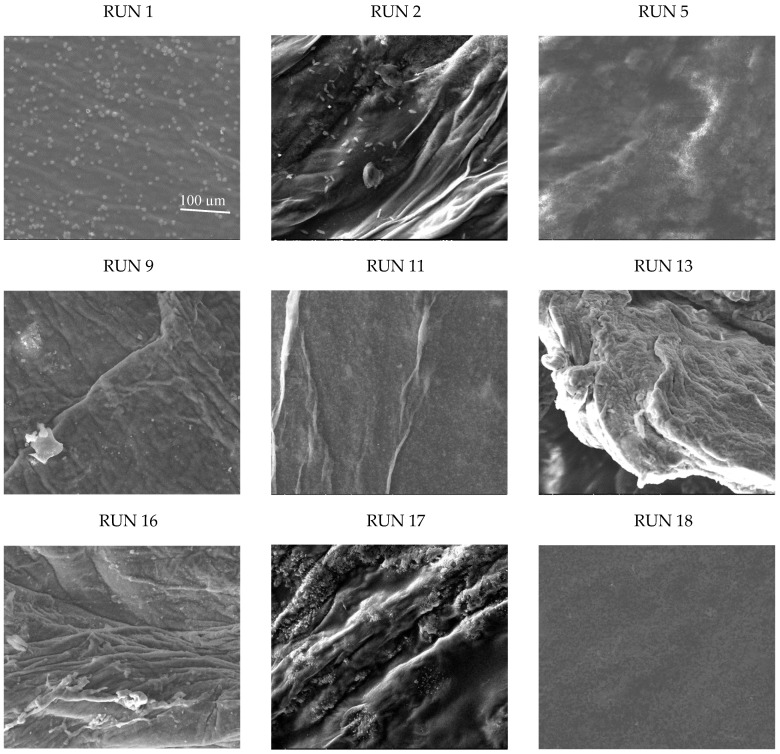
ESEM micrographs at 1000× (ETD detector) of the samples obtained from K2G44 functionalized with trough ex situ method (the scale bar indicated in the first micrograph can be applied for all the others).

In Figure A1 and Figure A2, the morphologies of all the samples obtained by the ex situ approach have been reported for BC produced by each strain. In particular, it can be noted that strong similarities are present for the samples produced in replicate (RUN2-RUN17; RUN11-RUN18; RUN13-RUN16 and RUN3-RUN4-RUN15), confirming the validity of the experimental procedure used to obtain the samples. Regarding other evaluations, most of the samples showed the expected morphology, having the appearance of fibers immersed in a homogeneous matrix. Depending on the angle of view, these fibers can also be seen in cross-section (e.g., RUN 1), and an estimation of the diameter of the BC fibers can be assessed at around 10 µm or less. In all the samples, no cluster or particle aggregation was detected. As an example, in Figure 1, the very homogeneous distribution of elemental Fe detected through EDS all over the RUN 5 sample is reported.

**Figure 2:**
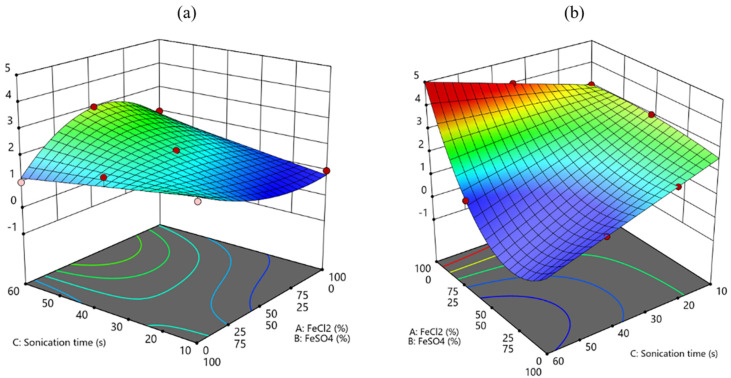
Contour plot for the response film quality: (**a**) K2G30 (**b**) K2G44.

**Figure 3:**
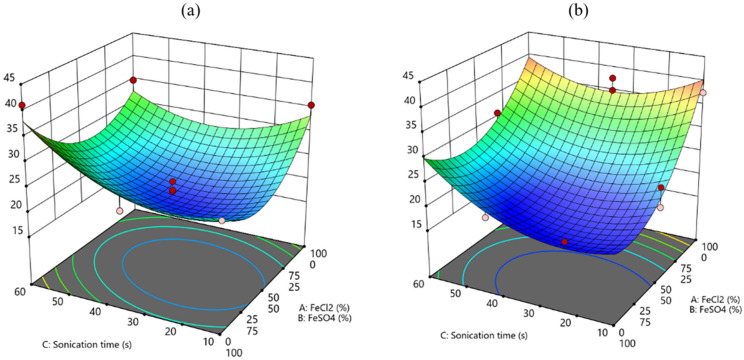
Contour plot for the response Fe %: (**a**) K2G30 (**b**) K2G44.

The statistical analysis (ANOVA) reported in Table 2 indicates that both the models regarding each response are significant (*p* < 0.05) with very good fitting parameters (R^2^ and Pred R^2^ > 0.75); thereafter, they can both be employed to assess the influence of the chosen factors and to draw predictions on the best condition to improve BC film quality and Fe content at the same time. Regarding the significant factors for each model, it can be assessed (Table 2) that all the chosen factors, single and in interaction, must be taken into account to better describe the final response in terms of film quality and Fe %. To better understand the influence of each factor, the model equations can be considered (Table A1), as well as the contour plots (Figure 2 and Figure 3) for each response.

Regarding the material quality, a relevant difference arises by considering the two bacterial strains, and depending on this variable, the others should be well-tailored to enhance the film quality. As shown in Figure 2, for both the bacterial strains, the highest sonication time should be employed (60 min) to promote the film quality, and this result was coherent with the fact that sonication has been included in this study to improve the homogenous distribution of the Fe particles and to avoid the formation of clusters and aggregates. Nevertheless, different proportions of the chemicals used for functionalization should be considered to improve one bacterial strain with respect to the other, as K2G30 film quality seems to be promoted by a 50:50 proportion of FeSO_4_ and FeCl_2_ (Figure 2a), whereas K2G44 was enhanced by the employment of only FeSO_4_ (Figure 2b).

Considering the Fe content (Figure 3), in similarity with the film quality, for both the bacterial strains a high sonication time should be employed, therefore confirming that sonication is a very powerful tool to promote an efficient distribution of the Fe particles, independently of the other considered factors. With respect to maximizing iron content, the results indicate that for strain K2G44, the use of FeCl_2_ alone is more effective than a mixed precursor solution, suggesting that combining FeCl_2_ and FeSO_4_ should be avoided (Figure 3b).

**Figure 4:**
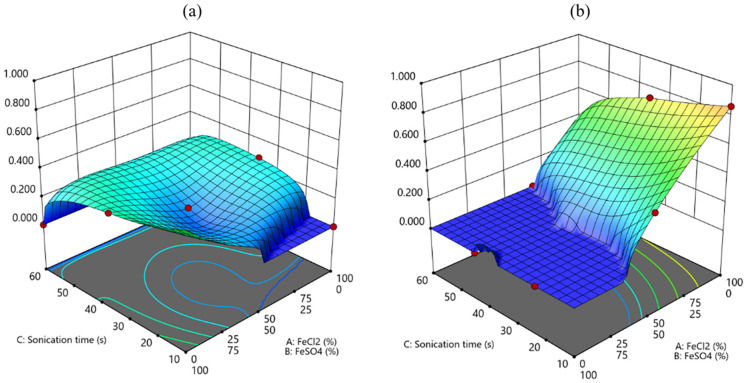
Contour plot for the Desirability function: (**a**) K2G30 (**b**) K2G44.

A desirability function has been implemented based on the results of the ANOVA analysis for both responses, with the aim of calculating the best overall condition to improve the film quality and the Fe % content. In particular, the objective of this function is to promote the highest possible film quality as well as Fe % content with one set of factors. The results of this function are represented in Figure 4, where some distinction must be considered depending on the bacterial strain, as the highest desirability (0.81) is reached for K2G44, indicating this bacterial strain is the most favorable for Fe functionalization in this condition. In addition, low sonication time and the only use of FeCl_2_ are the other conditions to improve both the film quality and the Fe content as calculated from this desirability function, which also considered the lowest possible sonication time as a requirement, to promote the highest efficiency of the method. For the bacterial strain K2G30 all the considered conditions are not suitable to reach a sufficient value of the desirability function that is quite stable at around 0.2–0.3 for all the combined factors.

**Figure 5:**
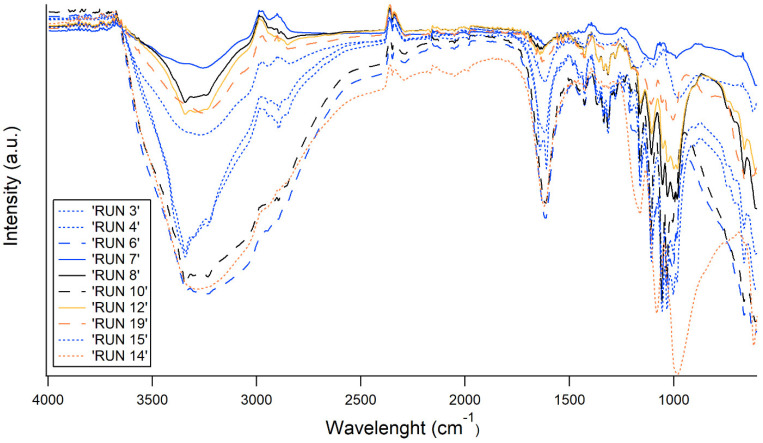
FT-IR spectra of samples obtained by K2G30 strain through ex situ approach. With the same color, the same sonication time is indicated (black = 10 min; blue = 35 min; and orange = 60 min). With the same line, the same proportion FeCl_2_:FeO_4_ is indicated (continuous line = 0:100; dashed line = 100:0 and point line = 50:50).

**Figure 6:**
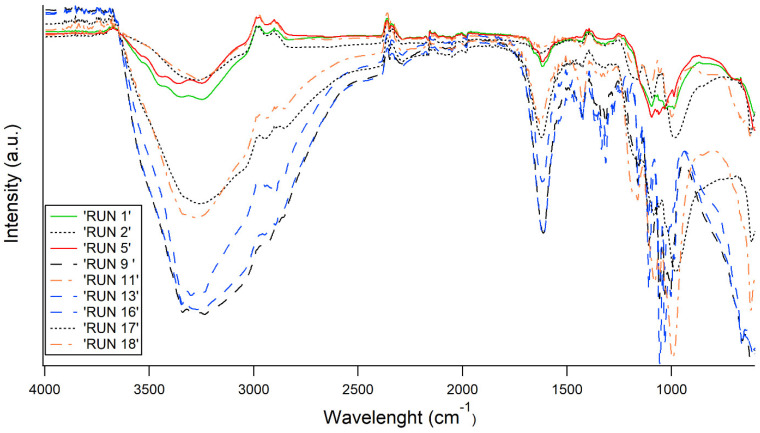
FT-IR spectra of samples obtained by K2G44 strain through ex situ approach. With the same color, the same sonication time is indicated (black = 10 min; red = 22 min; blue = 35 min; green = 43 min and orange = 60 min). With the same line, the same proportion FeCl_2_:FeO_4_ is indicated (continuous line = 0:100; dashed line = 100:0; point line = 50:50 and dashed-point line = 67:33).

The FT-IR spectra of all the samples are reported in Figure 5 and Figure 6, where all the runs were sorted depending on the bacterial strain, to improve the readability of the data. The main peaks are related to OH stretching vibrations in the region of 3350 cm^−1^, C-H stretching vibrations in the region near 3000 cm^−1^, and deformational vibrations of OH-groups of bound water at 1670 cm^−1^. Furthermore, the spectrum bands in the region of 1000–1200 cm^−1^ are related to stretching C–O–C and C–O vibrations. Nevertheless, the main structure is totally coherent with native BC, as discussed elsewhere by Gullo et al. [33] regarding the structure of the functionalized samples, as shown by the FT-IR. For both bacterial strains, differences arise mainly taking into account the proportion among FeCl_2_ and FeSO_4_ (different type of line), as this proportion mainly drives the stretching of the OH groups (3500–3000 cm^−1^) and O-related in general (1000–1200 cm^−1^). Instead, as expected, the sonication time (different color of the FT-IR spectra in Figure 5 and Figure 6) seems not to be related to any specific trend.

**Figure 7:**
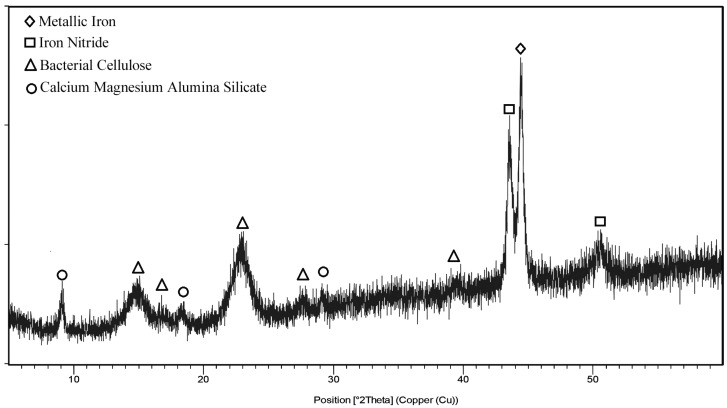
XRD spectra of RUN 9 sample.

In addition, regarding structure and considering the best sample in terms of the higher quantity of iron calculated by the desirability function, the microstructure of RUN 9 has been analyzed and reported (Figure 7). The XRD analysis detected metallic iron (ICDD: 01-085-1410—diamond in Figure 7), iron Nitride (ICDD: 01-075-2130—squares in Figure 7), BC (ICDD: 00-056-1718—triangles in Figure 7), and Calcium magnesium alumina silicate (ICDD: 00-025-0155—circles in Figure 7). The sample does not show any amorphous content in its structure, as any bump is present in the spectra. The same result can also be achieved by analyzing all the other RUNS of the experimental plan, as shown in Figure A5, demonstrating that the formation of these crystalline structures is mainly due to the application of the ex situ method described.

As shown in Table 3, all samples produced through the ex situ approach showed low resistivity and, therefore, conductive behavior. All values were around 1 MΩ, suggesting that the average conductivity is in the μS range. No strain-dependent trends were observed, suggesting the higher impact of the ex situ method on material conductivity. However, to properly estimate resistivity and conductivity, it would be necessary to measure geometrically identical samples to bypass any effect not related to the BC-Fe membrane composition. In fact, it is well known that as the thickness of the sample increases, a decrease in resistance, and thus an increase in electrical conductivity, is generally observed. In addition, the homogeneity of the microstructure of the sample (e.g., the presence of microporosity) may also have an influence on the electrical properties of the material, as less homogeneous structures are associated with longer electrical conductivity patterns and thus high resistivity. For these reasons, the resistivity values shown in Table 3 should be taken as indicative, and the method of sample production is currently under standardization. Nevertheless, these values are in good agreement with those reported previously for cellulose–nanocarbon composites and for low carbon nanotube content (0.4 wt%) [41,42]. Taking into account these preliminary results, a possible target application could be paper-based energy harvesters in nanogenerator devices.

### 3.2. In Situ Functionalization

The in situ functionalization was performed by cultivating bacterial strains for 4 days in media enriched with different concentrations of FeSO_4_-FeCl_2_ and supplemented solely with FeSO_4_ at different concentrations, not including FeCl_2_, due to the toxic effect of Cl- ions on bacteria [43]. At the end of the incubation time, cultures were sonicated and treated with NaOH, as reported by Sourty et al. [44]. During NaOH treatment, the BC pellicles gradually turned dark brown as a result of the precipitation of magnetic particles, which is in accordance with what was reported by Katepech et al. [23] and Chatimonth et al. [24].

**Figure 8:**
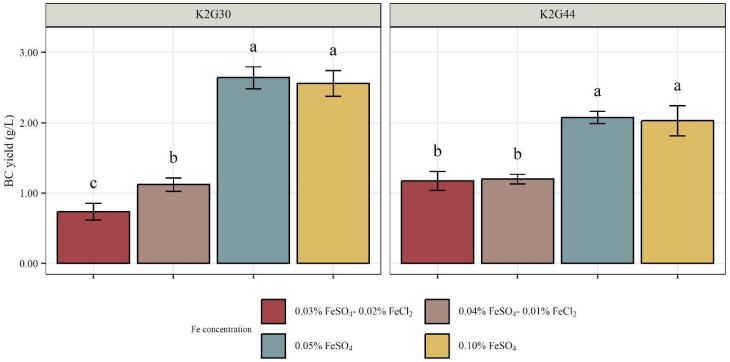
Quantification of BC (g/L) produced by K2G30 and K2G44 in Fe-enriched medium (0.03% FeSO_4_-0.02% FeCl_2_ *w*/*v*, 0.04% FeSO_4_-0.01% FeCl_2_ *w*/*v*, 0.05% FeSO_4_ and 0.10% FeSO_4_ *w*/*v*). Data are expressed as mean ± standard deviation (n = 4). Significant difference among BC yield is shown by different letters (*p* < 0.05).

Outcomes revealed lower BC yield for both K2G30 and K2G44 when FeCl_2_ was used in the medium formulation (Figure 8). K2G30 BC yield reached 0.74 ± 0.02 and 1.06 ± 0.01 g/L when cultivated in 0.03% FeSO_4_-0.02% FeCl_2_ *w*/*v* and 0.04% FeSO_4_-0.01% FeCl_2_ *w*/*v*, respectively. K2G44 had similar production (1.18 ± 0.02 g/L) in 0.04% FeSO_4_-0.01% FeCl_2_ *w*/*v*, but a higher one (1.09 ± 0.03 g/L) in 0.03% FeSO_4_-0.02% FeCl_2_ *w*/*v*.

On the other hand, in the presence of solely FeSO_4_, a high BC yield was obtained for both K2G30 and K2G44. The BC yield obtained by strain K2G30 was higher compared to that obtained by K2G44, independently of the concentration of FeSO_4_ supplemented (Figure 8). K2G30 BC yield exceeded 2.50 g/L, reaching 2.64 and 2.55 g/L in medium supplemented with 0.05% and 0.10% FeSO_4_, respectively. On the other hand, K2G44 BC yield reached 2.07 and 2.02 g/L.

The drastic reduction in BC yield occurring in the presence of FeCl_2_ confirmed that these media were unsuitable for producing ideal BC-based material through an in–situ approach. A partial solution could be increasing the incubation time to obtain thicker membranes, but the process would turn out to be unsustainable during a scale-up, resulting in an increase in production costs. For these reasons, only the materials obtained from cultivating K2G30 and K2G44 in media enriched solely with FeSO_4_ were characterized, evaluating their surface structure, chemical composition, and presence of Fe molecules.

**Figure 9:**
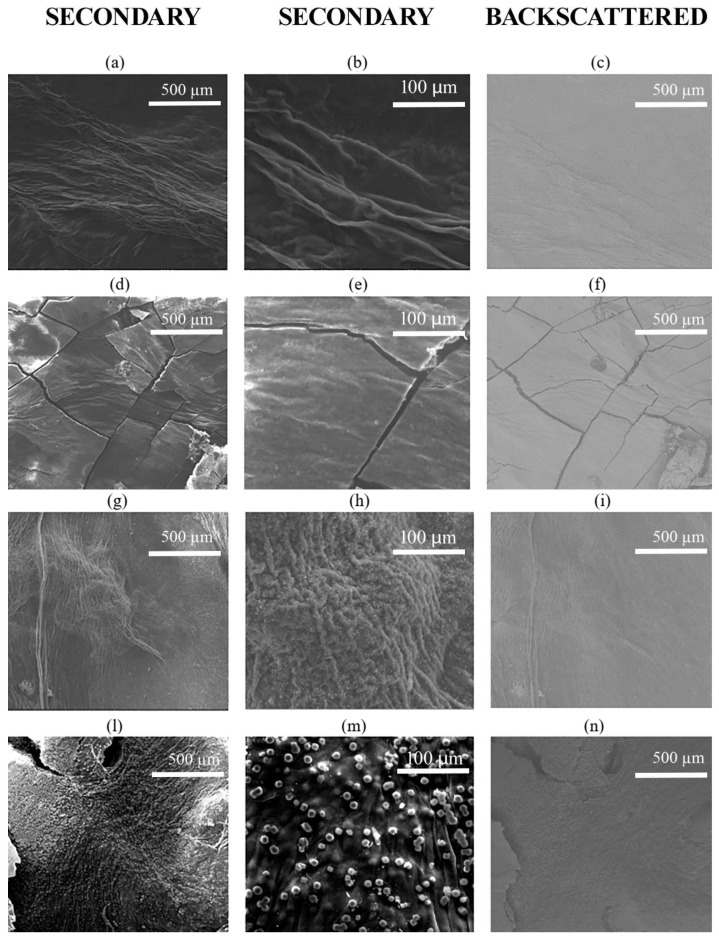
ESEM micrographs of samples K2G30 0.05% (**a**–**c**), K2G30 0.10% (**d**–**f**), K2G44 0.05% (**g**–**i**), K2G44 0.10% (**l**–**n**).

**Figure A3:**
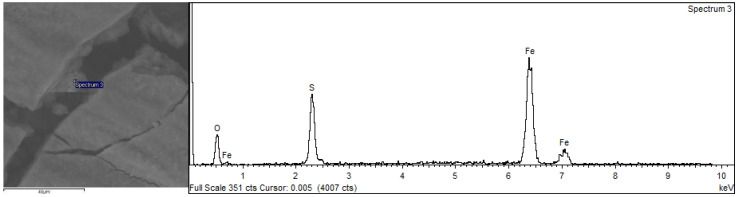
ESEM micrograph and EDS spectra at high magnitude (3000×) of the sample K2G30 0.10%.

**Figure A4:**
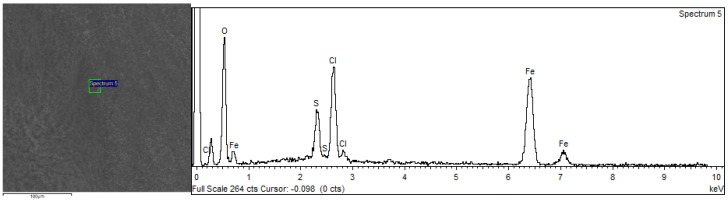
ESEM micrograph and EDS spectra of the sample K2G44 0.05% representative of 1 area of 5.

**Figure A5:**
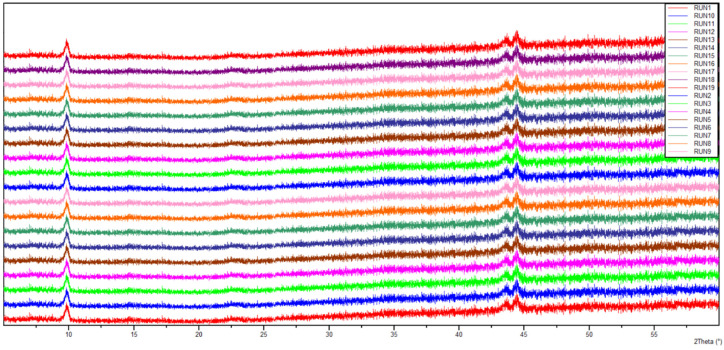
XRD spectra of all the samples obtained through the ex situ method.

In Figure 9, three micrographs have been reported for each sample obtained after the in situ functionalization, including backscattered and secondary electron signals and two different magnitudes for the latter, to better assess each sample morphology. A comparison with plain BC can be evaluated, considering previous results obtained without functionalization by Gullo et al. [33], where a very homogenous and smooth morphology was detected without cluster or secondary phase formation. ESEM micrographs suggest that, but not only, the bacterial strain has a key role in determining the morphology, as a smoother surface was observed for BC produced by the strain K2G30 (Figure 9a–f). Whereas BC obtained from K2G44 appeared more heterogeneous (Figure 9g–n). K2G44 is characterized by the presence of a spherical phase that has an increasing average diameter by moving from 0.05% to 0.10% of Fe compound concentration, resulting in more homogeneous dispersion in the sample K2G44 0.05% (Figure 9g–i). In particular, observing Figure 9h, the average diameter of the spherical phase in sample K2G44 0.05% is around 1 µm, and the average diameter in sample K2G44 0.10% is around 10 µm (Figure 9m). Regarding BC produced by K2G30, the variation in Fe compound concentration results only in a more brittle structure of the BC, as is clear from the fracture lines in Figure 9d–f related to the sample K2G30 0.10%. By observing this sample at high magnitude in cross-section (Figure A3) through these fracture lines, it is also possible to assess the presence of the functionalized structure (spherical phase homogeneously dispersed) related to a considerable Fe content below the sample surface, demonstrating the penetration capability of the functionalization process at least 20 µm below the surface. To complete the ESEM observations in Table 4, the EDS results have been reported for all the samples regarding the relevant area of each sample and, thereafter representative of the semi-quantitative chemical analysis for the samples. From these results, 3 of 4 combinations of bacterial strain-Fe concentration generated metallic particle functionalization as a relevant quantity of Fe was observed homogeneously dispersed in the BC matrix. Indeed, BC produced by K2G44 showed the presence of Fe in both layers produced in the media containing 0.05 and 0.10% *w*/*v* of FeSO_4_. On the other hand, for the functionalization of the BC produced by K2G30, a concentration of 0.05% *w*/*v* FeSO_4_ was not sufficient to detect any presence of Fe on the BC surface through EDS analysis. In fact, by also observing Figure 9 a–c, a morphology very close to plain BC can be observed. As expected, by increasing the Fe concentration in the broth culture, the Fe content in the BC increases from 6.06% to 8.42% for the bacterial strain K2G44. An example of the EDS spectra detected for 1 of 5 areas of the sample K2G44 0.05% has been reported in the Appendix A Figure A4.

**Figure 10:**
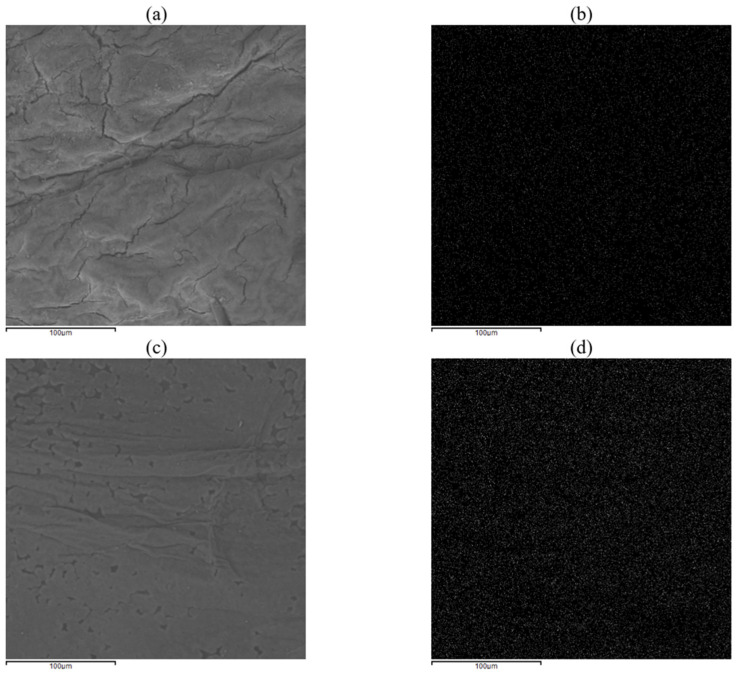
EDS elemental maps of samples K2G30 0.10% (**a**,**b**) and K2G44 K2G44 0.10% (**c**,**d**) related to the Fe element.

**Figure 11:**
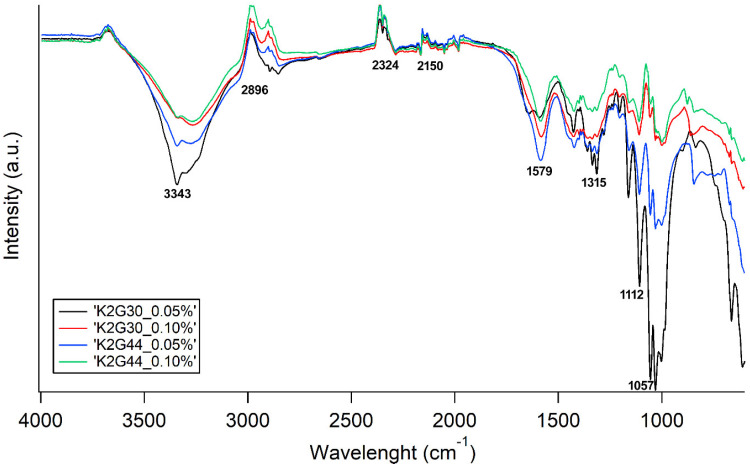
FT-IR spectra of bacterial cellulose samples obtained through an in situ approach.

To further assess the homogenous diffusion of Fe particles into the cellulose matrix, EDS elemental maps have been collected regarding Fe, demonstrating a homogenous presence of this element (white dots) all over the sample surface without cluster formation (Figure 10). The FT-IR analysis (Figure 11) is coherent with the findings of the FT-IR already reported in Figure 5 and Figure 6 and ESEM-EDS as the spectra related to the sample K2G30 0.05% differ from the others, therefore detecting a different structure due to missing Fe functionalization. Considering samples K2G30 0.10%, K2G44 0.05%, and K2G44 0.10% the bands representing -OH groups were observed to shift to a lower wavenumber position in the range 3020–3350 cm^−1^.

**Figure 12:**
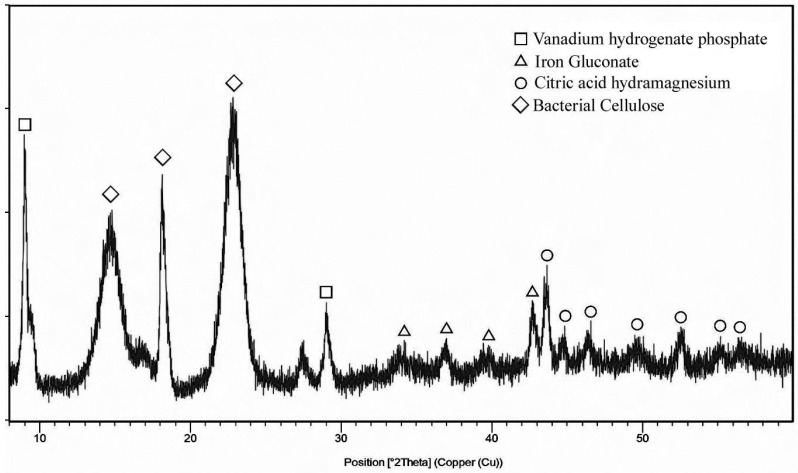
XRD spectra of K2G44 0.1% samples.

**Figure A6:**
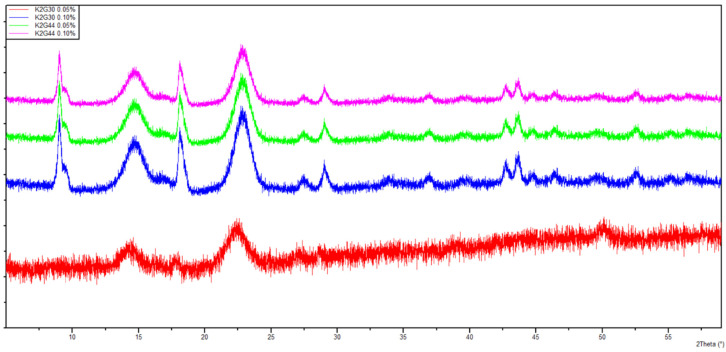
XRD spectra of all the samples obtained through the in situ method.

To complete the structural analysis, XRD analysis was performed on the sample with the higher Fe content detected by the EDS analysis, thereafter K2G44 0.10% (Figure 12). The founded crystalline phases were iron gluconate (ICDD: 00-005-0257—triangles in Figure 12), Citric Acid Hydrate (ICDD:00-004-0182—circles in Figure 12), Vanadium Hydrogenate Phosphate (ICDD: 00-043-0614—squares in Figure 12), and Bacterial Cellulose (ICDD: 00-056-1718—diamonds in Figure 12). Also, this sample did not show any particular amorphous content in its structure, as no bump is present in the spectra. The same results were observed in samples K2G44 0.05% and K2G30 0.05% (Figure A6). On the contrary, K2G30 0.05% showed only a few peaks related to BC crystalline structure (Figure A6), showcasing that the procedure conducted turned out to be not optimal in terms of sample functionalization. This result demonstrates that the formation of the crystalline structures reported in Figure 12 is mainly due to the application of the described in situ method.

This set of samples also exhibited conductive properties, as shown in Table 5. However, compared to the samples obtained by the ex situ approach, a marked increase in resistivity can be observed, also in terms of orders of magnitude, thus suggesting that the in situ approach was less effective. Data showed a strain-related influence on the resistivity of BC-Fe membranes. Indeed, samples obtained from K2G30 were slightly more conductive than those obtained from K2G44. As observed for the ex situ samples, even in this case any evaluation of possible trends will have to be assessed by comparing samples with the same size.

### 3.3. Acetic Acid Bacteria Strains Characteristics and Species Assignment

In this study, two acetic acid bacteria strains isolated from Kombucha tea and deposited at the UMCC culture collection [45,46] were assayed to assess the production of BC functionalized with FeSO_4_ and FeCl_2_ by an ex situ and in situ approach. The strains were selected based on our previous study in which the production of BC was tested in both a glucose medium and a glucose medium supplemented with ethanol. According to our studies, K2G44 produces BC in the range of 0.44 to 5.95 g/L, whereas K2G30 produces BC in the range of 0.74 to 23.20 g/L, depending on culture conditions. In addition, the strain K2G30, identified as *K. xylinus* was previously studied by whole genome analysis and metabolic traits of interest in producing high BC yield for different uses [33,47]. The strain can produce BC at high yield from different carbon sources, including glucose and mannitol [33,47]. Moreover, K2G30 was successfully tested for producing BC, which was immobilized in TiO_2_ and highly inorganic ceramic clay [48].

**Figure 13:**
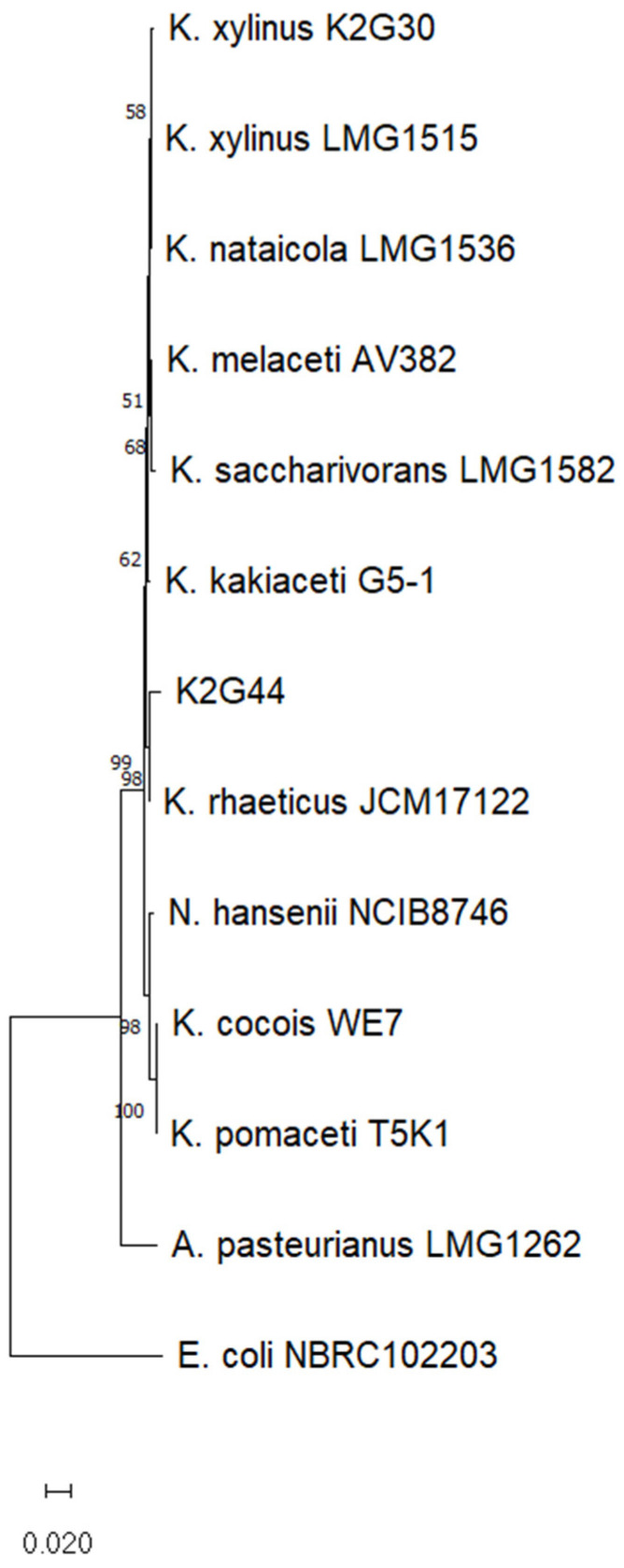
Notably, 16S rRNA neighbor-joining phylogenetic tree representing the phylogenetic distances of K2G44 and the type strain of nine *Komagataeibacter* species described as BC producers. The Tamura-Nei model was used to make the tree and a discrete Gamma distribution was used to model evolutionary rate differences among sites. Node numbers indicate bootstrap values obtained using 1000 replicates.

In the present study, we also used strain K2G44, a *Komagataeibacter* sp. strain. To infer the phylogeny of K2G44 (*Komagataeibacter* sp.), 9 sequences of *Komagataeibacter* species were used to cover most of the species described as BC producers. The K2G30 16S rRNA sequence was used as a reference for *K. xylinus* species. The differences in BC functionalization observed between the two bacterial strains are likely attributable to inherent species-specific properties. Variations in BC morphology can be linked to species-specific regulatory networks controlling cellulose biosynthesis, differences in the assembly of cellulose microfibrils into crystalline structures, and the differential expression of cellulose synthase genes, which influence the characteristics of BC [49,50,51]. Notably, the denser morphology observed in BC produced by K2G30 may restrict nanoparticle inclusion, whereas membranes with larger pores might exhibit a greater capacity for incorporating iron. The 16S rRNA sequences of the species *A. pasteurianus* LMG 1262 and *E. coli* NBRC 102203 were used as outgroups. The final total length of the K2G46 16S rRNA sequence resulted in 1348 bp. According to the clusterization shown in Figure 13, two main clusters could be identified. The first clade includes *Novacetimonas hansenii* NCIB 8746^T^ and one subclade, including *K. cocois* WE7^T^ and *K. pomaceti* T5K1^T^. The second clade groups most of the species, including *K. xylinus* K2G30, close to the *K. xylinus* LMG 1515^T^, and K2G44, grouped with *K. rhaeticus* JCM 17122^T^. As phylogenetic trees are hypotheses of evolutionary relationships, assessing the confidence of the phylogenetic tree, through techniques such as bootstrapping is crucial. In this study, the subclade of *K. rhaeticus* strains was supported by a bootstrap value of 100%. Between K2G44 and *K. rhaeticus* JCM 17122^T^, the similarity percentage resulted in 99.997%. Species characterization is a key factor in choosing bacterial strains for BC production. Besides the cultivation conditions (e.g., carbon sources), bacterial strains from different species may exhibit variations in metabolite production. Notably, both *K. xylinus* and *K. rhaeticus* species are known to produce gluconic acid, although the production levels can vary significantly between species [52,53]. BC membranes produced by different bacterial strains present different morphological characteristics, based on the differentiation of the regulatory networks controlling BC biosynthesis, which may differ from species to species, and the variations in the expression levels of BC synthase genes (involved in BC production and crystallization) [54,55]. Strains belonging to *K. xylinus* and *K. rhaeticus* species have been previously reported to grow on a wide range of carbon sources and to produce BC from several substrates [47,56,57,58,59,60]. In addition, both *K. xylinus* and *K. rhaeticus* strains have been previously used to produce metal-based electronically conductive BC [61,62,63].

## 4. Discussion

In this study, ex situ and in situ approaches were applied to produce Fe-enriched BC. The ex situ approach was developed through a statistical method, combining different FeSO_4_ and FeCl_2_ concentrations and sonication times. Through this statistical approach, mathematical models have been developed with predictive power to forecast the possible amount of Fe particles in the BC matrix depending on the ex situ conditions of functionalization. Regarding morphology and macroscopic properties, although the homogeneous dispersion of Fe atoms in the BC structure has been assessed for all the prepared specimens, two samples related to the bacterial strain K2G30 and functionalized by applying the ex situ method with a solution containing only FeSO_4_ resulted in a very brittle structure (RUN 8 and RUN 12) in strong similarity with the sample K2G30 0.10% obtained with the in situ method. This difference could be related to the higher BC amount produced by K2G30 compared to K2G44. The results are consistent with our previous studies reporting K2G30 as a high BC producer in different conditions of cultivation [37,47], highlighting AAB strains’ high variability in BC as previously observed [63]. The BC layer produced by K2G30 being thicker, it is reasonable to suppose a higher initial concentration of FeSO_4_ is needed to detect traces of Fe nanoparticles on BC layers. This result suggests the possibility of shortening the cultivation time for K2G30 to obtain a thinner BC layer suitable to be functionalized at lower FeSO_4_ concentrations, and this result is particularly important for the potential industrial scale-up. For all the samples, the achieved structural homogeneity suggests that sonication is effective for ensuring a uniform distribution of Fe particles—even in the shortest time (10 min). This finding also aligns with the optimal factor combination calculated from the desirability function (Figure 4). Furthermore, comparing the two precursors used for the two methods of functionalization, when FeCl_2_ was used in the medium formulation, the overall BC yield was lower, suggesting that chlorine acted as an inhibitor of bacterial cells’ growth. Indeed, chlorine damages the cell membrane by chlorinating the lipid-protein substance in the bacterial cell wall, forming toxic chloro-compounds, consequently inducing the leakage of macromolecules from the cells, which results in the disruption of the cell [64,65].

Molecular structure after functionalization: FT-IR spectra were mainly consistent with the typical bands already known for native BC [33] (Figure 5, Figure 6 and Figure 11). Regarding the modification of BC structure, in co-precipitation, the Fe^2+^ and Fe^3+^ ions are thought to anchor the cellulose -OH groups, causing a shift in the position of the -OH groups stretching vibration band [24,66]. Furthermore, FT-IR spectra of in situ functionalized samples (Figure 11) revealed distinct shifts in the O–H and C–O–C vibrational bands, consistent with the interaction of Fe^2+^ ions with cellulose hydroxyl groups. Such metal–BC interactions are well-documented and are known to influence the structural and functional properties of cellulose-based materials [67].

A similar mechanism accounts for the variation in peak intensity observed for other bands associated with oxygen-containing functional groups (1670 cm^−1^ and 1000–1200 cm^−1^), which, in our study, clearly distinguishes the K2G30 0.05% sample from all other samples. Regarding XRD analysis, first, it must be noted that there must be a substantial difference among the spectra obtained in this study and the plain BC [33], confirming the strong modification of the structure. The comparison among the XRD results obtained from the best sample of each method assessed that the in situ method (represented by sample K2G44 0.1%) provides the most closed bonding of iron with the BC matrix, as the greater part of the sample is identified as iron gluconate, also known as ferrous gluconate. This outcome is likely driven by the metabolic activity of acetic acid bacteria strains, which are known to oxidize glucose to gluconic acid via membrane-bound dehydrogenases. Gluconic acid, a chelating agent, can interact with Fe^2+^ ions in aqueous media, leading to the stabilization and subsequent incorporation of ferrous gluconate within the BC matrix. This mechanism is consistent with previous findings demonstrating the capacity of gluconic acid to form stable complexes with iron ions in solution [68]. The crystalline nature of this compound suggests that iron incorporation is not only surface-based but involves a bulk-phase modification of the BC structure.

Ferrous gluconate is a very popular food supplement as it is an affordable source of iron in the treatment of iron deficiency anemia. Furthermore, nevertheless, the elemental iron in the gluconate form (12%) is restrained with respect to other iron-based food supplements, such as ferrous sulfate (20%) or ferrous fumarate (33%); it has been demonstrated that it is very well absorbed by the stomach, reducing the critical gastric issue, easily arising during the treatment of iron deficiency [69,70]. In this context, the samples produced using the in situ method can be further evaluated for potential biomedical applications, such as the controlled release of iron in the human body, for example, through the fabrication of customized patches or bandages. On the other hand, by the ex situ method (sample RUN 9) a greater part of the iron was still in the metallic form. This result justifies the fact that a greater part of the iron was detected by the EDS, with respect to the in situ method, as the iron was present in the BC matrix in the metallic form. On the other hand, the in situ approach allowed the formation of a new crystalline phase, as the BC formation occurred in the culture broth containing iron. Other compounds, found both in ex situ and in situ methods, are mainly related to impurities or leftovers from the reagents used during the functionalization phases. It is also important to underline that, in any case, a totally crystalline structure was confirmed, as previously reported, BC functionalized with iron compounds [28,71]. Regarding conductive properties, we observed different resistivity (and thereafter, conductivity) according to the functionalization method used. In particular, with the ex situ method, a lower resistivity (~1 MΩ) and thereafter increased conductivity were obtained, potentially suitable for paper-based energy harvesting composites. This result was consistent with the XRD analysis, which revealed the presence of multiple iron-containing crystalline phases, including iron oxides and iron nitride, particularly in ex situ treated samples (e.g., RUN 9, Figure 7), confirming the oxidation of ferrous salts during the BC functionalization process. These findings indicate that Fe^2+^ ions were partially oxidized to Fe^3+^, likely driven by exposure to atmospheric oxygen and elevated temperatures during processing. The conversion of ferrous to ferric species under such conditions is a well-documented phenomenon and significantly impacts the physicochemical characteristics of the resulting composite materials [72,73]. Indeed, this oxidation is of particular relevance for materials intended for electronic applications, as the oxidation state and phase composition of iron directly influence magnetic behavior and electrical conductivity [74]. Finally, the absence of iron-glucose-derived compounds in ex situ samples supports a predominantly physical entrapment mechanism. The enhanced electrical conductivity measured (Table 3) is correlated to the increased Fe content and sharper XRD peaks in these samples, supporting the presence of conductive domains formed by metallic iron nanoparticles.

Based on previous literature, a first distinction must be made regarding the area of application of materials developed in this study. Regarding conductive BC-based materials, to date, no other studies are available using *Komagataibacter* sp. strains for producing BC functionalized by both in situ and ex situ methods.

Previous studies focused on a single bacterial strain rather than one production method [72]. In terms of resistivity and aiming to compare studies that utilized the same measurement apparatus, the results obtained in the present study are generally consistent with those reported in the literature. However, it should be noted that the incorporation of additional catalysts—such as pyrrole or polyaniline—has been shown to further enhance electrical conductivity [74,75]. Nonetheless, the absence of detailed information on sample size in many studies limits the objective comparisons of electronic properties. Conversely, other studies focus on the development of magnetic properties, which often involve the introduction of iron to synthesize Fe_3_O_4_ nanoparticles within the BC matrix or focus on enhancing energy harvesting properties [24,76,77].

## 5. Conclusions

In this study, BC produced by two AAB strains was successfully functionalized with FeCl_2_ and FeSO_4_ through both ex situ and in situ approaches. Differences in the composites obtained were observed and mathematically correlated with the specific bacterial strains. Based on these results, further investigations should be performed to evaluate the interactions and the effects of cultivating K2G30 in the presence of FeSO_4_, along with the possibility of reducing the incubation time to obtain a thinner and controlled layer easier to functionalize and, simultaneously, speed up the process by reducing the related costs. Overall the results of this study can be exploited to set up strategies for producing metal-based BC composites, contributing to expanding the feasibility of sustainable manufacturing processes in flexible electronics.

## Figures and Tables

**Table 1:** Complete experimental plan.

	Factors				Responses	
Run	A: FeCl_2_	B: FeSO_4_	C: Sonication Time	D: Strain	Film Quality	Fe
	%	%	min			%
1	0	100	43	K2G44	1	20.62
2	50	50	10	K2G44	3	24.49
3	50	50	35	K2G30	2	21.03
4	50	50	35	K2G30	2	20.78
5	0	100	22	K2G44	2	19.45
6	100	0	35	K2G30	1	22.76
7	0	100	35	K2G30	2	24.51
8	0	100	10	K2G30	2	26.92
9	100	0	10	K2G44	3	37.52
10	100	0	10	K2G30	1	35.52
11	67	33	60	K2G44	1	27.09
12	0	100	60	K2G30	1	41.08
13	100	0	35	K2G44	4	37.65
14	50	50	60	K2G30	3	23.85
15	50	50	35	K2G30	2	22.83
16	100	0	35	K2G44	4	34.98
17	50	50	10	K2G44	3	20.34
18	67	33	60	K2G44	1	30.84
19	100	0	60	K2G30	2	34.78

**Table 2:** ANOVA results where A = FeCl_2_; B = FeSO_4_; C = Sonication time; D = Strain.

Response	*p*-Value	Significant Factors	R^2^	Pred—R^2^
Film quality	<0.0001	A; B; C; D; AB; AC; AD; BC; BD; ABC; ABD; ACD; BCD; ABCD	0.93	0.85
Fe %	0.0013	A; B; C; D; AB; AC; AD; BC; BD; CD; C^2^	0.87	0.77

**Table 3:** Resistivity values of all the samples obtained through the Ex situ approach.

Sample	Resistivity (MΩ)	Sample	Resistivity (MΩ)
RUN1	1.30 ± 0.05	RUN11	0.34 ± 0.04
RUN2	0.56 ± 0.03	RUN12	1.23± 0.05
RUN3	0.76 ± 0.07	RUN13	0.45 ± 0.03
RUN4	1.34 ± 0.04	RUN14	0.43 ± 0.06
RUN5	0.98 ± 0.05	RUN15	0.43 ± 0.06
RUN6	0.45 ± 0.08	RUN16	0.24± 0.05
RUN7	0.43 ± 0.10	RUN17	1.45 ± 0.10
RUN8	0.45 ± 0.08	RUN18	1.15 ± 0.10
RUN9	0.43 ± 0.06	RUN19	1.32± 0.05
RUN10	0.78 ± 0.08		

**Table 4:** Semi-quantitative chemical analysis of the samples obtained by in situ approach (Atomic %).

Sample	C (%)	O (%)	Na (%)	P (%)	Fe (%)	Cl (%)	S (%)	Others * (%)
K2G30 0.05%	46.43	45.52	6.94	0.55	-	-	-	0.36
K2G30 0.10%	13.33	70.55	-	-	6.97	-	8.98	0.17
K2G44 0.05%	35.56	52.98	-	-	6.06	3.86	1.54	-
K2G44 0.10%	14.19	68.97	-	-	8.42	1.90	6.52	-

* Others are elements that have been found in traces, having an atomic % equal to or below the instrumental detectability limit (0.5%).

**Table 5:** Resistivity values of all the samples obtained through Ex situ approach.

Sample	Resistivity (MΩ)
K2G30 0.05%	35 ± 4
K2G30 0.10%	27 ± 3
K2G44 0.05%	95 ± 7
K2G44 0.10%	55 ± 5

## Data Availability

Data associated with this study have been deposited into the public repository Genbank under the accession number PP755334.

## Appendix A

**Table A1:** Equations of the calculated models.

Response	Equation
Film quality	= +0.633333 × FeCl_2_ +2.36667 × FeSO_4_ −3.60000 × FeCl_2_ × FeSO_4_ +0.020000 × FeCl_2_ × Sonication time −0.020000 × FeSO_4_ × Sonication time +0.160000 × FeCl_2_ × FeSO_4_ × Sonication time (Strain = K2G30) = +2.60000 × FeCl_2_ +3.08000 × FeSO_4_ +3.12000 × FeCl_2_ × FeSO_4_ +0.040000 × FeCl_2_ × Sonication time −0.048000 × FeSO_4_ × Sonication time −0.232000 × FeCl_2_ × FeSO_4_ × Sonication time (Strain = K2G44)
Fe %	= +38.53330 × FeCl_2_ +30.29563 × FeSO_4_ −0.001430 × Sonication time −30.15726 × FeCl_2_ × FeSO_4_ −0.600468 × FeCl_2_ × Sonication time −0.370344 × FeSO_4_ × Sonication time +0.008329 × Sonication time^2^ (Strain = K2G30) +45.39840 × FeCl_2_ +22.13853 × FeSO_4_ +0.001430 × Sonication time −30.15726 × FeCl_2_ × FeSO_4_ −0.600468 × FeCl_2_ × Sonication time −0.370344 × FeSO_4_ × Sonication time +0.008329 × Sonication time^2^ (Strain = K2G44)
